# Combination of the ankle-brachial index and percentage of mean arterial pressure to improve diagnostic sensitivity for peripheral artery disease: An observational study

**DOI:** 10.1097/MD.0000000000012644

**Published:** 2018-09-28

**Authors:** Han-Wei Lin, I-Te Lee

**Affiliations:** a Division of Endocrinology and Metabolism, Department of Internal Medicine, Taichung Veterans General Hospital, Taichung City; b School of Medicine, National Yang-Ming University, Taipei City; c School of Medicine, Chung Shan Medical University; d College of Science, Tunghai University, Taichung, Taiwan.

**Keywords:** ankle-brachial index, percentage of mean arterial pressure, peripheral artery disease, sensitivity

## Abstract

The ankle-brachial index (ABI) is a noninvasive method for screening for peripheral artery disease (PAD). However, false-negative findings of the ABI may limit its clinical use. The percentage of mean arterial pressure (%MAP) calculated from pulse volume recording has been reported to predict all-cause mortality. We hypothesized that the %MAP would be helpful to screen for PAD in subjects with a normal ABI. We examined whether using a combination of the ABI and %MAP would provide greater diagnostic sensitivity for PAD than using the ABI alone.

In this cross-sectional study, we retrospectively reviewed the medical records of patients who had undergone multiple detector computed tomography (MDCT) angiography of the lower extremities following measurement of the ABI with pulse volume recording. PAD was diagnosed based on MDCT angiography.

A total of 215 lower extremities of 114 patients were included in our analyses. An optimal cut-off %MAP value of 42.5% was used to diagnose PAD based on MDCT in patients with an ABI > 0.90. Using a combination of an ABI < 0.90 and a %MAP ≥ 42.5% as diagnostic criteria for PAD resulted in better sensitivity (76.9%) than using the ABI alone (56.5% for an ABI < 0.90 and 63.4% for an ABI < 1.00). Using logistic regression analysis, we found that patients having both an ABI < 0.90 and an ABI > 0.90 with a %MAP ≥ 42.5% had a significantly higher risk of PAD than those having an ABI > 0.90 with a %MAP < 42.5% (odds ratio = 7.165, *P* = .006; odds ratio = 12.544, *P* < .001; respectively).

Both the sensitivity and specificity were better when using a combination of an ABI ≤ 0.90 and a %MAP ≥ 42.5% than when using a low or borderline ABI. The %MAP is helpful for PAD screening in subjects with an ABI > 0.90.

## 1 Introduction

Peripheral artery disease (PAD), a sequential result of atherosclerosis, has become a heavy public health burden.^[1,2]^ Although intermittent claudication is a typical symptom of PAD, many patients remain asymptomatic.^[3]^ The ankle-brachial index (ABI) is a useful noninvasive screening tool for PAD.^[4]^ PAD diagnosed based on an ABI ≤ 0.90 had similar risks of all-cause mortality, myocardial infarction, and stroke as symptomatic PAD.^[5]^

However, PAD diagnosis using an ABI ≤ 0.90 has a reported sensitivity of only 76% in Chinese patients,^[6]^ and the sensitivity may decrease in specific populations, such as the elderly and diabetics.^[7,8]^ To increase the diagnostic sensitivity for PAD, the threshold of the ABI value should be revised. Several articles have reported that subjects with borderline ABI values between 0.91 and 0.99 showed higher risks of PAD and all-cause mortality than those with ABI values ≥ 1.00.^[9,10]^

Pulse volume recording can easily be automatically obtained for ABI assessment.^[11]^ The percentage of mean arterial pressure (%MAP) and the upstroke time (UT) calculated from pulse volume recording were significantly higher in patients with PAD than those without.^[12]^ It has been reported that the combined parameters of ABI, %MAP, and UT provide better sensitivity than the definition of an ABI ≤ 0.99.^[13]^ However, the specificity was relatively decreased after adding %MAP and UT to the diagnostic criteria of PAD.

An ABI ≤ 0.90 was reported to be a strong predictor of mortality, and a high %MAP provided an additional risk for mortality in patients with an ABI > 0.90; however, subjects with an ABI ≤ 0.90 might still have a higher risk of mortality than those with an ABI > 0.90 and a high %MAP.^[11]^ In the present study, we attempted to apply the same 2-step assessment: first, by screening based on an ABI ≤ 0.90 in all patients, followed by screening of the %MAP in patients with an ABI > 0.90. We examined the diagnostic accuracy of this 2-step assessment compared to that of using the ABI alone.

## 2 Materials and methods

### 2.1 Subjects

This case–control study was conducted at the Division of Endocrinology and Metabolism in Taichung Veterans General Hospital. We retrospectively reviewed the medical records of patients who had undergone multiple detector computed tomography (MDCT) for angiography of their lower extremities between June 2009 and September 2017. We excluded enrolled patients who did not undergo pulse volume recording assessment of the ABI prior to MDCT for the same indication. Subjects with an ABI > 1.40 or end-stage renal disease were excluded. Demographic characteristics and laboratory data were collected. The study protocol was approved by the Institutional Review Board of Taichung Veterans General Hospital, Taichung, Taiwan before data collection.

### 2.2 Procedures

The ABI and %MAP were measured simultaneously using a validated automatic device (VP-1000; Colin Corporation, Hayashi, Komaki City, Japan). Cuffs were placed on the arms and ankles on both sides, and were connected to both a plethysmographic sensor that detected volume change and an oscillometric pressure sensor to measure blood pressure. Patients underwent this assessment after resting in the supine position for at least 5 minutes. The higher systolic blood pressure of the 2 arms was selected as the brachial pressure. The right and left ABI values were determined by dividing the systolic pressure in each ankle by the brachial pressure. In addition, the %MAP was determined based on the ankle pulse volume waveforms. The %MAP indicates the height of the mean area of the arterial wave divided by the peak amplitude. The reproducibilities of the ABI and %MAP were examined in a group of 20 subjects. Highly linear correlations of the ABI (*r* = 0.90, *P* < .001) and %MAP (*r* = 0.73, *P* < .001) between the results of the first and second measurements were observed. Based on the Bland–Altman plots, the 95% confidence intervals (CIs) were 0.02 ± 0.01 for the bias of the ABI and −0.33 ± 0.67 for the %MAP between repeated measurements.

Angiography was performed with the thinnest collimation and a reconstruction thickness of 2.5 mm using a 64-detector computed tomography scanner (Brilliance 64; Philips Healthcare, Best, The Netherlands). Angiography included the common iliac artery, external iliac artery, internal iliac artery, femoral artery, popliteal artery, anterior tibial artery, posterior tibial artery, peroneal artery, and dorsalis pedis artery in the arterial phase scan after an intravenous administration of 1.3 mL/kg (of body weight) of contrast medium followed by a 30-mL saline chaser. PAD was defined as an arterial lesion with luminal narrowing ≥70%.

### 2.3 Statistical analyses

Among the 114 enrolled patients, 13 lower extremities were excluded from the analyses owing to a history of surgical or vascular intervention. Continuous data are presented as the mean ± standard deviation, and categorical data are presented as numbers (percentages). A chi-squared test was used to detect statistical differences in the categorical variables. An independent sample *t* test was used to detect the intergroup statistical difference in the continuous variables. A receiver operating characteristic (ROC) curve was used to determine the optimal cut-off point of the %MAP for PAD in patients with an ABI > 0.9. Multivariate linear regression analysis was used to analyze the associated factors for the MDCT-based PAD diagnosis. The estimated glomerular filtration rate (eGFR) was calculated using the following formula: eGFR (mL/min/1.73 m^2^) = 186 × (serum creatinine [mg/dL])^−1.154^ × (age [year])^−0.203^ (× 0.742, if female), based on the Modification of Diet in Renal Disease equation. A value of *P* < .05 was considered statistically significant. All analyses were performed using SPSS version 22.0 software (International Business Machines Corp, New York, NY).

## 3 Results

**Figure 1:**
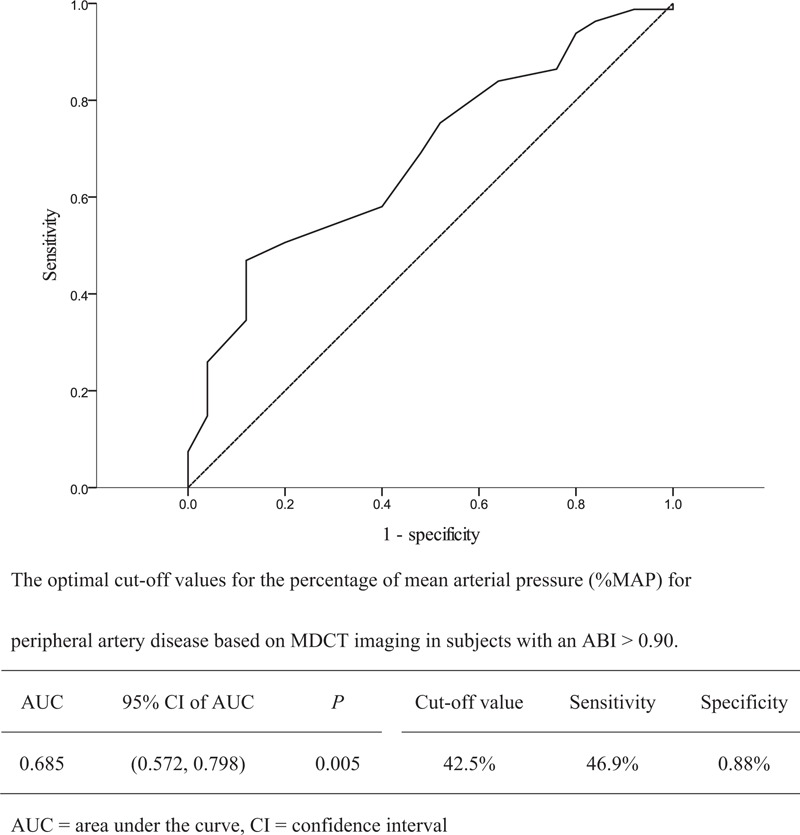
Receiver operating characteristic curve analysis to determine the cut-off level of the percentage of mean arterial pressure (%MAP) for diagnosis of peripheral artery disease (PAD) based on multiple detector computed tomography (MDCT) angiography.

A total of 215 lower extremities of 114 patients were included in the analyses. The median duration between assessments of the ABI and MDCT was 15 days (interquartile range, 4–56 days). Based on MDCT diagnosis, 186 extremities were placed into the MDCT(+) group and 29 extremities into the MDCT(−) group. The proportion of male patients was lower in the MDCT(+) than in the MDCT(−) group (50.5% vs. 79.3%, *P* = .007), and the proportion of patients using an angiotensin-converting enzyme (ACE) inhibitor or angiotensin II receptor antagonist (ARB) was higher in the MDCT(+) than in the MDCT(−) group (62.9% vs. 37.9%, *P* = .019). A lower mean ABI value (0.86 ± 0.25 vs. 1.07 ± 0.14, *P* < .001) and a higher %MAP (46.2 ± 6.2 vs. 39.5 ± 4.1%, *P* < .001) were observed among patients in the MDCT(+) group than among those in the MDCT(−) group (Table 1). However, only 105 (56.5%) patients with an ABI value ≤ 0.90 were in the MDCT (+) group. In the MDCT(+) group, a lower %MAP was observed in the patients with an ABI > 0.90 than in those with an ABI ≤ 0.90 (42.1 ± 4.7 vs. 49.3 ± 5.4%, *P* < .001; Table 1). Using ROC curve analysis, a cut-off value of 42.5% for the %MAP provided better prediction for PAD diagnosed by MDCT in patients with an ABI > 0.90 (Fig. 1).

**Table 1:**
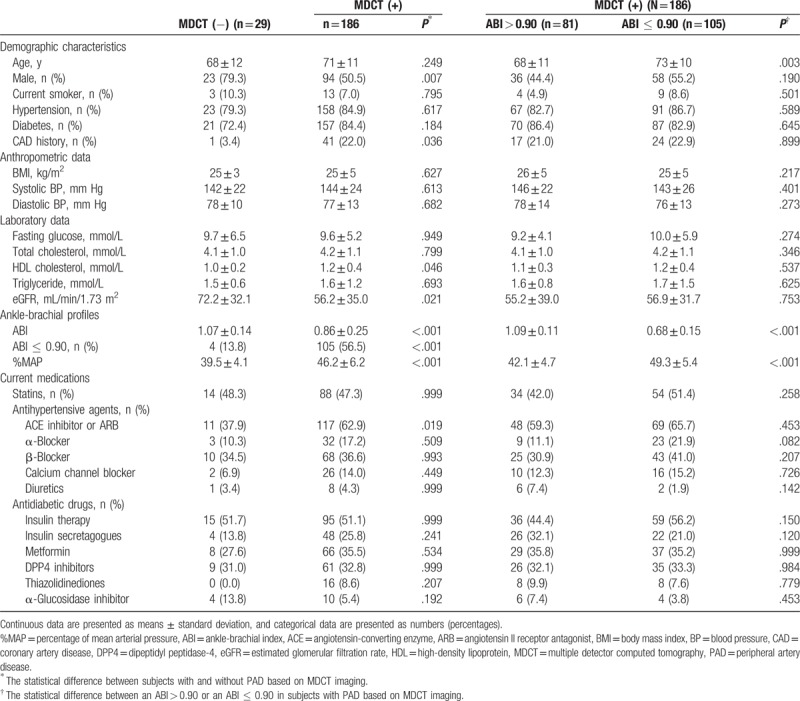
Characteristics of patients with and without PAD, and of those with an ABI > 0.90 and ≤0.90 in those with PAD, based on MDCT angiography.

**Figure 2:**
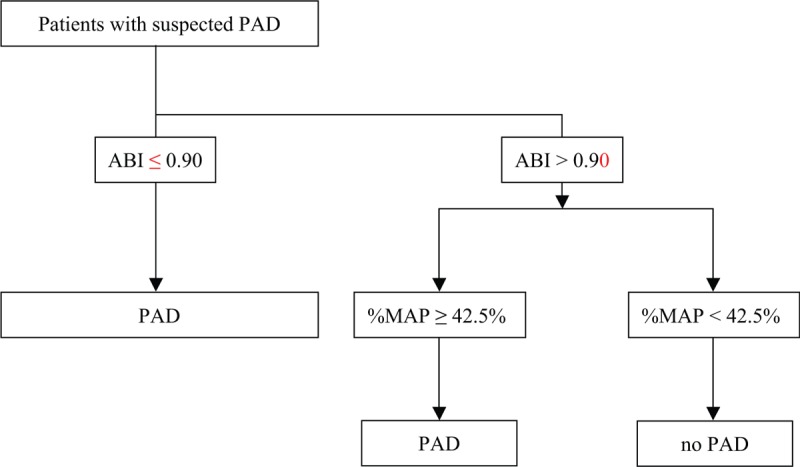
Flow chart for subjects who underwent measurement of the ankle-brachial index (ABI) and percentage of mean arterial pressure (%MAP) to diagnose peripheral artery disease (PAD).

To increase sensitivity, we attempted to elevate the cut-off value of the ABI to 1.00, and while the criterion of an ABI ≤ 1.00 had a sensitivity of 63.4%, the specificity was reduced to 69.0%. Conversely, we used an ABI ≤ 0.90 as the first-step criterion for PAD diagnosis, then used a %MAP ≥ 42.5% as the second criterion (Fig. 2). This 2-step criteria for diagnosing PAD provided a sensitivity of 79.6% and specificity of 75.9%, which were both better than those provided by using an ABI ≤ 1.00 (Table 2).

**Table 2:**
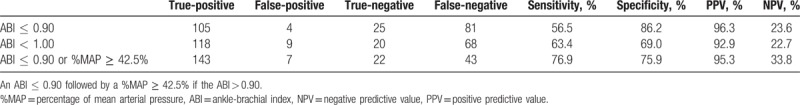
Diagnosis of peripheral artery disease based on different criteria.

Logistic regression analysis revealed that patients with an ABI < 0.90 were significantly associated with having PAD diagnosed via MDCT (odds ratio: 12.544, 95% CI: 3.751–41.949; *P* < .001), and patients with an ABI > 0.90 and a %MAP ≥ 42.5% were also significantly associated with having PAD diagnosed via MDCT (odds ratio: 7.165, 95% CI: 1.746–29.405; *P* < .006) after adjustment for age, sex, high-density lipoprotein cholesterol, eGFR, and current ACE inhibitor or ARB use (Table 3).

**Table 3:**
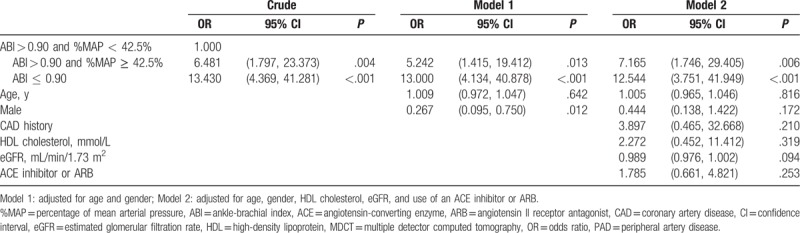
Logistic regression analysis showing the associated factors for PAD based on MDCT.

## 4 Discussion

Using a %MAP ≥ 42.5% provided additional benefit for PAD screening in patients with an ABI > 0.90. Based on the report from the American College of Cardiology/American Heart Association Task Force on Clinical Practice Guidelines, a borderline ABI is reported to be associated with PAD and mortality.^[14]^ However, despite increasing the ABI threshold value to 1.00 for PAD diagnosis, our results showed that only using a combination of an ABI ≤ 0.90 and a %MAP ≥ 42.5% provided better sensitivity and specificity than using an ABI < 1.00.

In line with our findings, Hashimoto et al^[13]^ reported that using a combination of an ABI < 1.00, %MAP ≥ 45%, and UT ≥ 180 milliseconds provided better sensitivity than using an ABI < 1.00 alone. However, the specificity using the combination criteria was obviously less than that using the ABI alone (60.0% vs. 84.4%).^[13]^ In the present study, the %MAP was significantly lower in subjects with an ABI > 0.90 than in those with an ABI ≤ 0.90. Our strength in the present analysis was to select the optimal cut-off point of the %MAP in subjects with an ABI > 0.90, instead of in all subjects, because the purpose for using the %MAP was to select PAD patients from subjects with an ABI > 0.90. Using the combination criteria of an ABI ≤ 0.90 and a %MAP ≥ 42.5%, both the sensitivity and specificity were better than those using the criterion of an ABI < 1.00 alone.

In a Korean study, the sensitivity and specificity of using an ABI < 0.90 was reported as 61% and 87%, respectively, for PAD diagnosis; however, the sensitivity may have been lower due to arterial stiffness, especially in elderly or diabetic populations.^[15]^ The waveform of pulse volume recording includes an upstroke with a sharp peak, followed by a downstroke with a dicrotic notch. A flattened waveform with a delayed upstroke will be observed in an occluded artery.^[16]^ Therefore, a high %MAP reflecting a flattened arterial wave can be a sign of arterial occlusion, and provide a diagnostic criterion for PAD in subjects with falsely elevated ABI values due to noncompressible vessels.^[17–19]^

Several methods, including the exercise test and toe-brachial index, are suggested for PAD screening in high-risk patients with an ABI > 0.90.^[20–22]^ Given recent technical advances, pulse volume recording can be simultaneously and automatically collected during ABI measurement. The presence of arterial stiffness may not influence pulse volume recording because the pulsatile pressure waveform is generated based on volume change, instead of pressure profiles, detected by the transducer after venous compression by pneumatic cuffs.^[23]^ Furthermore, the %MAP has also been reported to be a useful predictor for critical limb ischemia and all-cause mortality.^[11,24]^

Diabetes is an important risk factor for PAD.^[25]^ The prevalence of diabetes is reported to be approximately 50% in patients with suspected PAD in teaching hospitals.^[26]^ In the present study, 84.4% of patients with PAD diagnosed based on MDCT had known diabetes. This high proportion of patients with diabetes may be owing to the availability of ABI and %MAP assessment, which were performed at the Division of Endocrinology and Metabolism in our hospital.

There were some limitations of our study. First, we did not include subjects with an ABI > 1.40, and the diagnostic value of the %MAP could not be applied to this population. Similarly, MDCT was performed for subjects with suspected PAD in our clinical practice. Therefore, our findings in this retrospective study may not be applicable to the general population. Second, we did not further assess the difference in PAD risk between subjects with an ABI ≤ 0.90, and those with an ABI > 0.90 and a %MAP ≥ 42.5%. Although the combination of using the ABI and %MAP increases the sensitivity for the diagnosis of PAD, subjects with an ABI ≤ 0.90 may have a higher PAD risk than those with an ABI > 0.90, even with a %MAP ≥ 42.5%. Third, to simplify the screening criteria, we only used the %MAP and ABI values based on our previous findings.^[11]^ We did not include the UT and pulse wave velocity in our analysis, which could also be collected during ABI measurement.

In conclusion, using a combination of an ABI ≤ 0.90 and a %MAP ≥ 42.5% can provide greater sensitivity and specificity for the diagnosis of PAD than using the criteria of a low (≤0.90) or borderline (0.91–0.99) ABI.

## Author contributions

**Data curation:** Han-Wei Lin.

**Formal analysis:** I-Te Lee.

**Methodology:** I-Te Lee.

**Supervision:** I-Te Lee.

**Writing – original draft:** Han-Wei Lin.

**Writing – review & editing:** I-Te Lee.
